# Cancer-specific survival in patients with cholangiocarcinoma after radical surgery: a Novel, dynamic nomogram based on clinicopathological features and serum markers

**DOI:** 10.1186/s12885-023-11040-9

**Published:** 2023-06-12

**Authors:** Shurui Zhou, Yue Zhao, Yanzong Lu, Weiling Liang, Jianmin Ruan, Lijun Lin, Haoming Lin, Kaihong Huang

**Affiliations:** 1 Department of Gastroenterology, Zhongshan School of Medicine, Sun Yat-sen Memorial Hospital, Sun Yat- sen University, The 107th of Yanjiang West Road, Guangzhou, 510120 China; 2 Department of Ophthalmology, No.903 Hospital of PLA Joint Logistic Support Force, Hangzhou, 310013 China; 3 Department of Pancreatobiliary Surgery, Sun Yat-sen Memorial Hospital, Sun Yat-sen University, The 107th of Yanjiang West Road, Guangzhou, 510120 China

**Keywords:** Cholangiocarcinoma, Nomogram, Surgery, Prognosis

## Abstract

**Background:**

This study aims to (1) identify preoperative testing-based characteristics associated with enhanced prognosis and survival for cholangiocarcinoma patients, and (2)create a distinctive nomogram to anticipate each patient’s cancer-specific survival (CSS).

**Methods:**

Retrospective analysis was performed on 197 CCA patients who underwent radical surgery at Sun Yat-sen Memorial Hospital; they were divided into a 131-person “training cohort” and a 66-person “internal validation cohort.“ The prognostic nomogram was created following a preliminary Cox proportional hazard regression search for independent factors influencing the patients’ CSS. Its applicable domain was examined via an external validation cohort, which included 235 patients from the Sun Yat-sen University Cancer Center.

**Results:**

The median follow-up period for the 131 patients in the training group was 49.3 months (range, 9.3 to 133.9 months). One-, three-, and five-year CSS rates were 68.7%, 24.5%, and 9.2%, respectively, with the median CSS length being 27.4 months (range: 1.4 to 125.2 months). PLT, CEA, AFP, tumor location, differentiation, lymph node metastasis, chemotherapy, and TNM stage were determined to be independent risk factors for CCA patients by univariate and multivariate Cox proportional hazard regression analysis. We were able to accurately predict postoperative CSS after incorporating all of these characteristics into a nomogram. The AJCC’s 8th edition staging method’s C-indices were statistically substantially (P < 0.001) lower than the nomogram’s C-indices (0.84, 0.77, and 0.74 in the training, internal and external validation cohorts respectively).

**Conclusions:**

A realistic and useful model for clinical decision-making and the optimization of therapy is presented as a nomogram that includes serum markers and clinicopathologic features for predicting postoperative survival in cholangiocarcinoma.

**Supplementary Information:**

The online version contains supplementary material available at 10.1186/s12885-023-11040-9.

## Background

The epithelial cells that line the bile ducts are the likely origin of cholangiocarcinoma (CCA), which is a malignant disease characterized by occult onset, rapid progress, a high relapse rate, and high mortality [1, 2]. Nearly 3% of all gastrointestinal cancers are caused by cholangiocarcinoma, making it the second most common primary liver cancer, and various epidemiological studies have shown a dramatic rise in morbidity and death globally over the last few years [3]. Surgical resection for CCA remains the only curative treatment modality at present, but the prognosis after surgery is still poor [4, 5].

The gold standard for determining the prognosis of CCA is the 8th iteration of the American Joint Committee on Cancer tumor node metastasis (AJCC-TNM) staging system [6]. However, the system’s predictive ability is limited by the fact that CCA is very heterogeneous and, as a consequence, individuals with the same TNM stage and treatment outcomes have varying prognoses. Moreover, a practical and effective predicting system is lacking based on objective indicators specifically formulated for postoperative prognosis. Therefore, the ability to identify survival in patients with CCA following radical resection that relies on the development of independent prognostic markers and determine the optimal personalized therapeutic strategy for postoperative treatment options was identified.

In comparison with the traditional staging system, the nomogram has undoubtable advantages in the aspect of sensitivity and specificity, and thus, it has been proposed as a potential alternative for prediction in the majority of cancer types [7–9]. Therefore on this basis, we tried to construct a novel prognostic model for CCA using a nomogram approach by combining clinicopathological features, systemic inflammation indicators and serum tumor markers. To further investigate whether the novel nomogram can more accurately evaluate prognosis, we compared its predictive performance and clinical applicability to that of the standard TNM staging system protocol.

Accumulating evidence suggests that active inflammation is closely related to carcinogenesis, which has a significant effect on cancer development and progression [10, 11]. Peripheral blood cell level indicates inflammation response, which is considered an important indicator in tumor progression and regarded as reliable and legible clinical prognostic markers in several kinds of cancer [12, 13]. Moreover, several tumor biomarkers, such as carcinoembryonic antigen (CEA), alpha-fetoprotein (AFP), as well as carbohydrate antigen 19 − 9 (CA 19 − 9), are applied in facilitating early diagnosis, reflecting the state of illness, and evaluating postsurgical follow-up of gastrointestinal tumor [14–16].

Generally speaking, our research aims to develop a unique prognostic model that incorporates inflammatory and tumor signs to reliably predict survival in patients with CCA receiving the primary surgical intervention.

## Methods

### Patients recruitment

We conducted a retrospective study carried out at the Sun Yat-sen Memorial Hospital, in which CCA patients underwent radical resection from January 2009 to January 2019. Inclusion criteria included the following: (1) no history of other malignant diseases, (2) CCA diagnosis confirmed by histopathology, and (3) after the radical operation. Exclusion criteria included the following: (1) tumors of other origin or metastatic liver tumor, (2) preoperative chemoradiotherapy or other adjuvant chemoradiotherapy, (3) perioperative mortality (death during hospitalization or within 30 days of the operation) [17, 18], and (4) inflammatory diseases, active infection, or immunocompromised status not related to cancer within 1 month before blood examination. Finally, overall enrollment in the major cohort trial was 197, with patients split evenly between a training cohort (n = 131) and an internal validation group (n = 66) using a randomization schedule.

Patients with CCA who had radical resection at the Sun Yat-sen University Cancer Center were recruited retrospectively from January 2009 to January 2019 to constitute the external validation group, which used the same study selection (inclusive criteria and exclusive criteria) as described above.

The retrospective study complied with the rules of research ethics and it was approved by our hospital’s ethics committee.

*Data collection*.

Baseline personal information from each study participant was obtained in each cohort by medical records. The clinicopathologic features were assessed from clinical records. The disease diagnoses were established by clinicians, who analyzed all information including laboratory exams and image tests. For discharged patients, clinical prognostic data were gathered by medical records review, telephone interviews, or personal visits. Follow-up was conducted every six months after resection. Our main analytic outcome was cancer-specific survival (CSS), between the start of surgery and the patient’s first cancer-related death is known as the postoperative phase or the end of follow-up.

*Study variables*.

Preoperative serum markers (i.e., testing results collected from preoperative assessments) obtained within 2 weeks before the surgery was inquired from the electronic medical records and clinical laboratory findings of the hospital information system. The study variables contained inflammatory markers such as the level of hemoglobin (HGB), leukocyte (WBC), neutrophil (NEUT), lymphocyte (L), and platelet (PLT); and tumor biomarkers, such as CEA, AFP, CA 19 − 9 and et al. The AJCC TNM classification (8th edition)^6^ was used as the only basis for staging tumors.

### *Statistical* analysis

The software involved in this study to perform statistical analysis contained SPSS 22.0 software (Chicago, USA), Stata 15.0 software (StataCorp LP, USA), and R statistical language (R packages, version 4.0.2) [19]. When comparing two continuous variables with the same distribution, for statistical analysis, we employed the Student’s t-test and the Mann-Whitney U test where the distribution was not normal. The categorical data were also examined using the chi-square or Fisher exact test. It was assumed that a difference would be statistically significant if P < 0.05.

The optimal cutoff point of continuous variables in our novel nomogram was identified using operating characteristic (ROC) curve analyses, X-tile software (version 3.6.1, USA) [20], and the split method according to the nomogram score. CSS analysis was plotted using the Kaplan-Meier technique, and comparisons were made using the log-rank test. Further, we performed both univariate and multivariate analyses using the Cox proportional hazards regression model to calculate hazard ratios (HR) and 95% confidence intervals (CI).

Using R software and the RMS tool to analyze the data, a unique nomogram was developed based on the presence of many independent risk variables. More importantly, we established a dynamic nomogram to provide visualized risk prediction [21]. As a means of contrasting our nomogram with existing staging systems [22], we performed regression analysis using the rcorrp. cens in hmisc in R to generate the calibration curve and get the C-index. To evaluate the nomogram’s accuracy in making predictions, it was subjected to internal and external validation using the same statistical techniques as the training group. The prediction sensitivity, accuracy, and clinical utility of our nomogram were compared to those of the conventional staging method using both time-dependent receiver operating characteristic (ROC) [23] curve analysis and decision curve analysis (DCA) [24].

## Results

### Patient characteristics and clinicopathological data of CCA patients

In total, 197 patients who satisfied the standards stipulated by the inclusion criteria were entered into our primary cohort from Sun Yat-sen Memorial Hospital lasted from the years 2009 to 2019, which were randomized to a training cohort (n = 131) as well as an internal validation cohort (n = 66) in a ratio of 2:1. Description and analysis of CCA patients’ baseline characteristics of two cohorts are represented in Table 1. Among the 131 samples in the primary training cohort, there were respectively 80 (61.1%) men and 51 (38.9%) women with a median age of 59 years old. Moreover, the median follow-up time in our primary training cohort was 49.3 months (range, 9.3 to 133.9 months). The statistical study revealed a median CSS duration of 27.4 months (range: 1.4–125.2) and 1-year, 3-year, and 5-year CSS rates of 68.7%, 24.5%, and 9.2%, respectively.

**Table 1:** The clinical and pathological characteristics of cholangiocarcinoma patients from the main training cohort, internal validation cohort, and external validation cohort, respectively

Variables	Primary Cohort (n = 131)		Internal Validation Cohort (n = 66)		External Validation Cohort (n = 235)	
	No. of Patients	%	No. of Patients	%	No. of Patients	%
Age						
≤ 60	67	51.1	35	53	149	63.4
>60	64	48.9	31	47	86	36.6
Gender						
Male	80	61.1	38	57.6	142	60.4
Female	51	38.9	28	42.4	93	39.6
History of hepatitis						
Yes	54	38.9	28	42.4	NA	NA
No	80	61.1	38	57.6	NA	NA
HGB(g/L)						
≤ 154.5	125	95.4	62	93.9	NA	NA
>154.5	6	4.6	4	6.1	NA	NA
WBC(*10^9/L)						
≤ 5.57	26	19.8	15	22.7	NA	NA
>5.57	105	80.2	51	77.3	NA	NA
NEUT(*10^9/L)						
≤ 4.35	59	45.0	20	30.3	NA	NA
>4.35	72	55.0	46	69.7	NA	NA
L (10^9/L)						
≤ 3.12	126	96.2	97	147	NA	NA
>3.12	5	3.8	3	4.5	NA	NA
PLT(*10^9/L)						
≤ 245.5	63	48.1	24	36.4	125	53.2
>245.5	68	51.9	42	63.6	110	46.8
AFP(µg/L)						
≤ 11.8	126	96.2	62	93.9	205	87.2
>11.8	5	3.8	4	6.1	30	12.8
CEA(µg /L)						
≤ 8.0	104	79.4	53	80.3	201	85.5
>8.0	27	20.6	13	19.7	34	14.5
CA 19 − 9(U/mL)						
≤ 125.4	54	41.2	28	42.4	NA	NA
>125.4	77	58.8	38	57.6	NA	NA
TBil(µmol/L)						
≤ 18.9	46	35.1	28	42.4	NA	NA
>18.9	85	64.9	38	57.6	NA	NA
DBil(µmol/L)						
≤ 4.4	36	27.5	22	33.3	NA	NA
>4.4	95	72.5	44	66.7	NA	NA
ALT(U/L)						
≤ 44	58	44.3	31	47	NA	NA
>44	73	55.7	35	53	NA	NA
AST(U/L)						
≤ 111	109	83.2	45	68.2	NA	NA
>111	22	16.8	21	31.8	NA	NA
GGT(U/L)						
≤ 138	47	35.9	25	37.9	NA	NA
>138	84	64.1	41	62.1	NA	NA
ALP(U/L)						
≤ 90	19	14.5	14	21.2	NA	NA
>90	112	85.5	51	77.3	NA	NA
ALB(g/L)						
≤ 34.3	28	21.4	15	22.7	NA	NA
>34.3	103	78.6	51	77.3	NA	NA
GLB(g/L)						
≤ 31.6	85	64.9	37	56.1	NA	NA
>31.6	46	35.1	29	43.9	NA	NA
Tumor location						
iCCA	71	54.2	42	63.6	140	59.6
eCCA	60	45.8	24	36.4	95	40.4
Tumor size(cm)						
≤ 5	89	67.9	37	56.1	164	69.8
>5	42	32.1	29	43.9	71	30.2
Tumor number						
Single	108	82.4	54	81.8	NA	NA
Multiple	23	17.6	12	18.2	NA	NA
Tumor differentiation						
Well	34	26.0	15	22.7	68	28.9
Low-moderate	97	74.0	51	77.3	167	71.1
TNM stage						
I	25	19.1	7	10.6	91	38.4
II	39	29.8	22	33.3	66	27.8
III+IV	67	51.1	37	56.1	80	33.8
Lymph node metastasis						
Yes	49	37.4	28	42.4	92	39.1
No	82	62.6	38	57.6	143	60.8
Nerve invasion						
Yes	55	42.0	30	45.5	NA	NA
No	76	58.0	36	54.5	NA	NA
Vascular invasion						
Yes	38	29.0	19	28.8	NA	NA
No	93	71.0	47	71.2	NA	NA
Chemotherapy						
Yes	49	37.4	26	39.4	99	42.1
No	82	62.6	40	60.6	136	57.9

Notes: HGB, hemoglobin; WBC, leukocyte; NEUT, neutrophil; L, lymphocyte; PLT, platelet; AFP, α-fetoprotein; CA19-9, carbohydrate antigen 19 − 9; CEA, carcinoembryonic antigen; DBIL, direct bilirubin; TBIL, total bilirubin; ALT, alanine aminotransferase; ALP, alkaline phosphatase; AST, aspartate aminotransferase; GGT, γ-glutamyltransferase; ALB, albumin; GLB, globulin; TNM, tumor–node–metastasis, TNM stage according to AJCC (American Joint Committee on Cancer) eighth edition; NA, not applicable

### Factors associated with CCA patient survival independently predicting CSS in the initial training cohort

The results of univariate and multivariate studies of the possible relationships between clinicopathological characteristics and cancer-specific survival are reported in Table 2. Univariate analyses of 131 CCA patients in the training cohort suggested that leukocyte count, neutrophil count, platelet count, serum CA19-9, AFP, CEA, ALP (alkaline phosphatase), GLB (globulin), tumor location, tumor size, tumor number, tumor differentiation, TNM stage, lymph node metastasis, vascular invasion and chemotherapy were significantly related to CSS. The platelet count, serum AFP, CEA, tumor location, tumor differentiation, lymph node metastasis, chemotherapy, and TNM stage were all shown to be significant independent risk factors for CCA patients in a multivariate analysis. Additional Figure S1 displays the Kaplan-Meier survival curves based on these non-TNM prognostic criteria.

**Table 2:** Univariate and multivariate analysis for cancer-specific survival of patients with cholangiocarcinoma in the training cohort

Variables	Univariate analysis		Multivariate analysis	
	HR (95% CI)	*P*-value	HR (95% CI)	*P*-value
Age (>60/≤60)	1.178(0.745–1.861)	0.483		
Gender (Female/ Male)	1.370(0.858–2.188)	0.188		
History of hepatitis (Yes/ No)	1.227(0.772–1.951)	0.386		
HGB(g/L) (>154.5/≤154.5)	1.351(0.492–3.708)	0.559		
WBC(*10^9/L) (>5.57/≤5.57)	3.284(1.504–7.710)	0.003	1.360(0.507–3.647)	0.541
NEUT(*10^9/L) (>4.35/≤4.35)	2.083(1.281–3.388)	0.003	0.955(0.462–1.976)	0.902
L(*10^9/L) (>3.12/≤3.12)	1.343(0.422–4.274)	0.618		
PLT(*10^9/L) (>245.5/≤245.5)	2.184(1.353–3.526)	0.001	3.946(2.155–7.225)	<0.001
AFP(µg/L) (>11.8/≤11.8)	6.239(2.395–16.250)	<0.001	3.572(1.151–11.085)	0.028
CEA(µg/L) (>8.0/≤8.0)	4.019(2.447-6.600)	<0.001	2.590(1.246–5.384)	0.011
CA19-9(U/mL) (>125.4/≤125.4)	2.012(1.212–3.341)	0.007	1.565(0.865–2.831)	0.138
TBil(µmol/L) (>18.9/≤18.9)	1.344(0.817–2.209)	0.244		
DBil(µmol/L) (>4.4/≤4.4)	1.301(0.756–2.240)	0.342		
ALT(U/L) (>44/≤44)	1.260(0.789–2.011)	0.333		
AST(U/L) (>111/≤111)	1.390(0.785–2.459)	0.258		
GGT(U/L) (>138/≤138)	1.534(0.934–2.519)	0.091		
ALP(U/L) (>90/≤90)	2.333(1.067–5.099)	0.034	2.222(0.871–5.674)	0.095
ALB(g/L)(>34.3/≤34.3)	1.226(0.691–2.396)	0.426		
GLB(g/L) (>31.6/≤31.6)	1.977(1.244–3.140)	0.004	1.135(0.648–1.989)	0.659
Tumor location (iCCA/eCCA)	1.645(1.028–2.634)	0.038	2.261(1.064–4.803)	0.034
Tumor size(cm) (>5/≤5)	2.207(1.384–3.518)	0.001	1.523(0.748–3.103)	0.246
Tumor number (Multiple/ Single)	3.929(2.287–6.751)	<0.001	1.469 (0.774–2.788)	0.239
Tumor differentiation (Low-moderate/Well)	1.903(1.085–3.338)	0.025	2.118(1.051–4.271)	0.036
TNM stage				
I	ref.	<0.001	ref.	0.013
II	5.245(1.776–15.490)	0.003	4.600(1.385–15.277)	0.013
III+IV	10.212(3.660-28.491)	<0.001	6.214(1.830-21.104)	0.003
Lymph node metastasis (Yes/No)	4.469(2.762–7.232)	<0.001	2.934(1.470–5.855)	0.002
Nerve invasion (Yes/No)	1.091(0.684–1.739)	0.714		
Vascular invasion (Yes/No)	1.715(1.063–2.768)	0.027	1.180(0.670–2.080)	0.567
Chemotherapy (Yes/No)	2.075(1.264–3.406)	0.004	3.185(1.745–5.813)	<0.001

Notes: HGB, hemoglobin; WBC, leukocyte; NEUT, neutrophil; L, lymphocyte; PLT, platelet; CEA, carcinoembryonic antigen; AFP,α-fetoprotein; CA19-9, carbohydrate antigen 19 − 9; DBIL, direct bilirubin; TBIL, total bilirubin; AST, aspartate aminotransferase; ALT, alanine aminotransferase; GGT, γ-glutamyltransferase; ALP, alkaline phosphatase; GLB, globulin; ALB, albumin; TNM, tumor–node–metastasis, TNM stage according to AJCC (American Joint Committee on Cancer) 8th edition; HR, hazard ratio; CI, confidence interval

### A prognostic nomogram for CCA patients’ cancer-specific survival

**Fig. 1:**
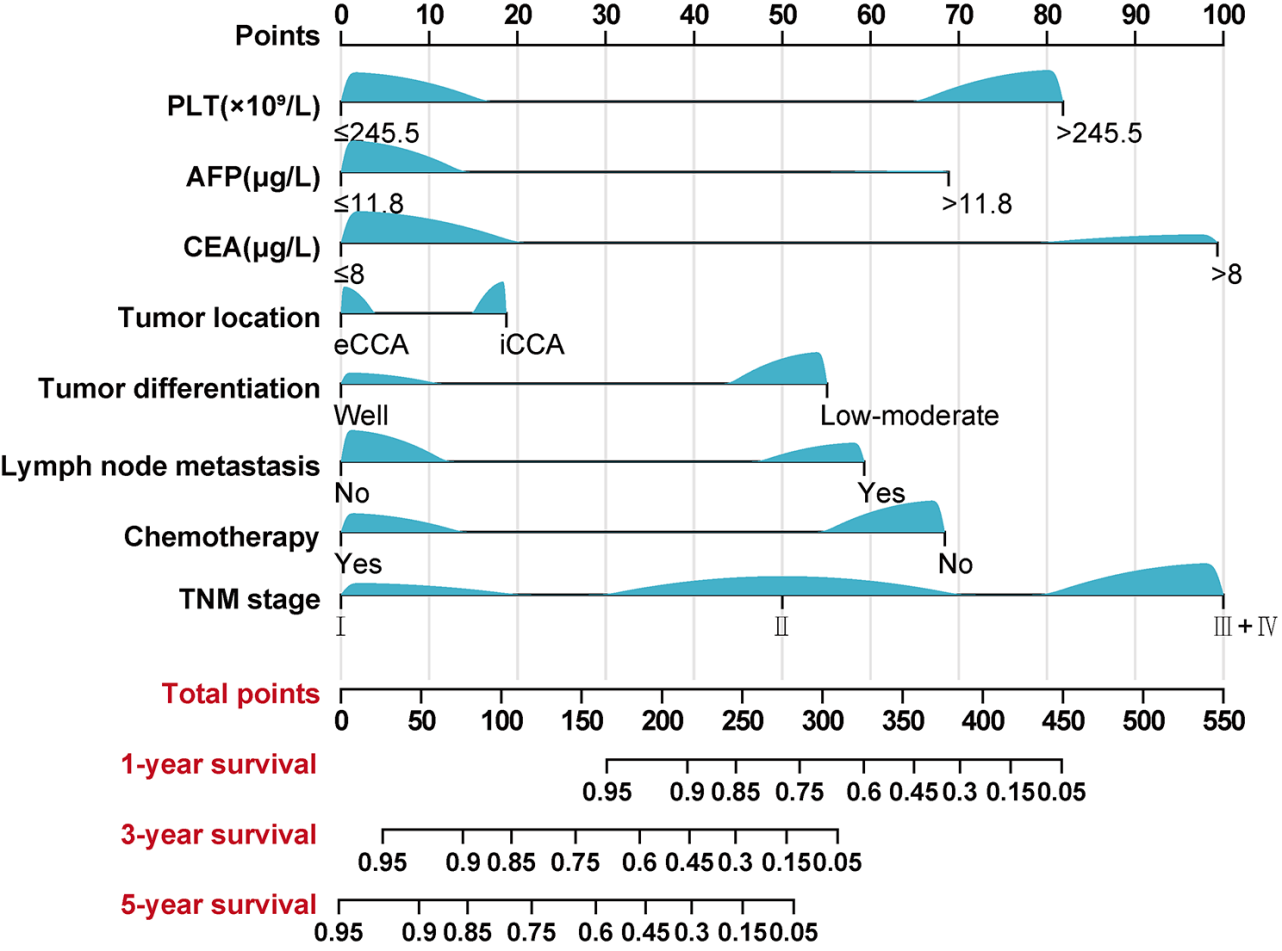
Nomogram predicting 1-year, 3-year, and 5-year cancer-specific survival for patients with cholangiocarcinoma

**Fig. 2:**
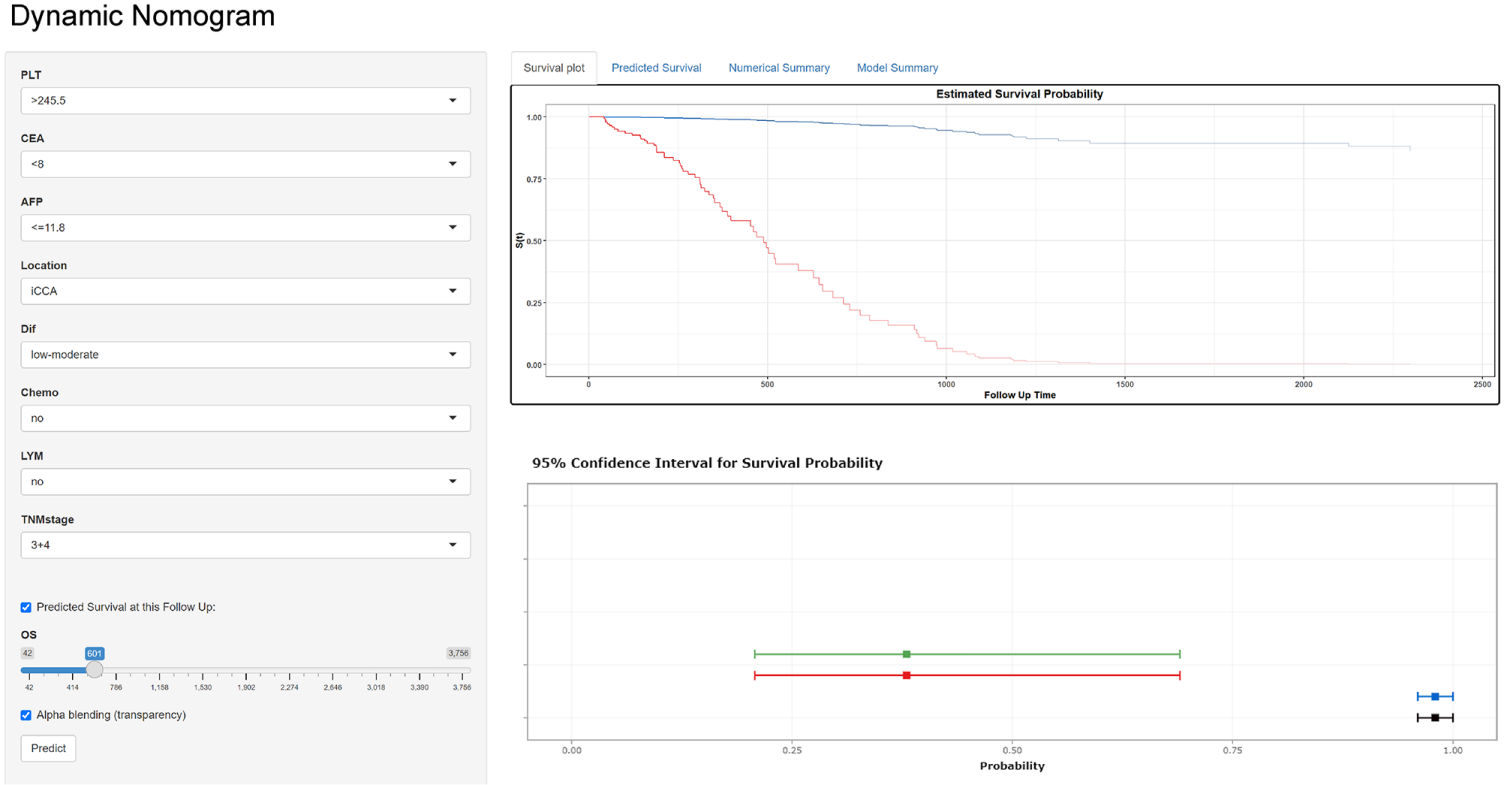
Dynamic nomogram (online version) for patients with cholangiocarcinoma that predicts survival unique to malignancy

Following this research, we developed a unique nomogram to predict CSS in postoperative CCA patients by including the discovered independent risk variables (Fig. 1). As indicated in the top line of Fig. 1, we can convert each independent risk variable to a point value for each patient. For example, the PLT point value was 82 when the PLT level of the patient was higher than 245.5 × 10^9^/L. Then each point value of 8 variables was summed up to calculate a total point as risk scores, which was able to predicate the probability of survival at 1, 3, and 5 years as shown in the bottom of Fig. 1. A web-based and easily accessible dynamic nomogram is also performed in Fig. 2, which can be accessed online to give assistance to clinicians along with researchers. It was a very easy and convenient way to obtain predicted survival probability across time of CCA patients, by which clinicians input clinical characteristics first and then acquire the results produced from the webserver.

**Fig. 3:**
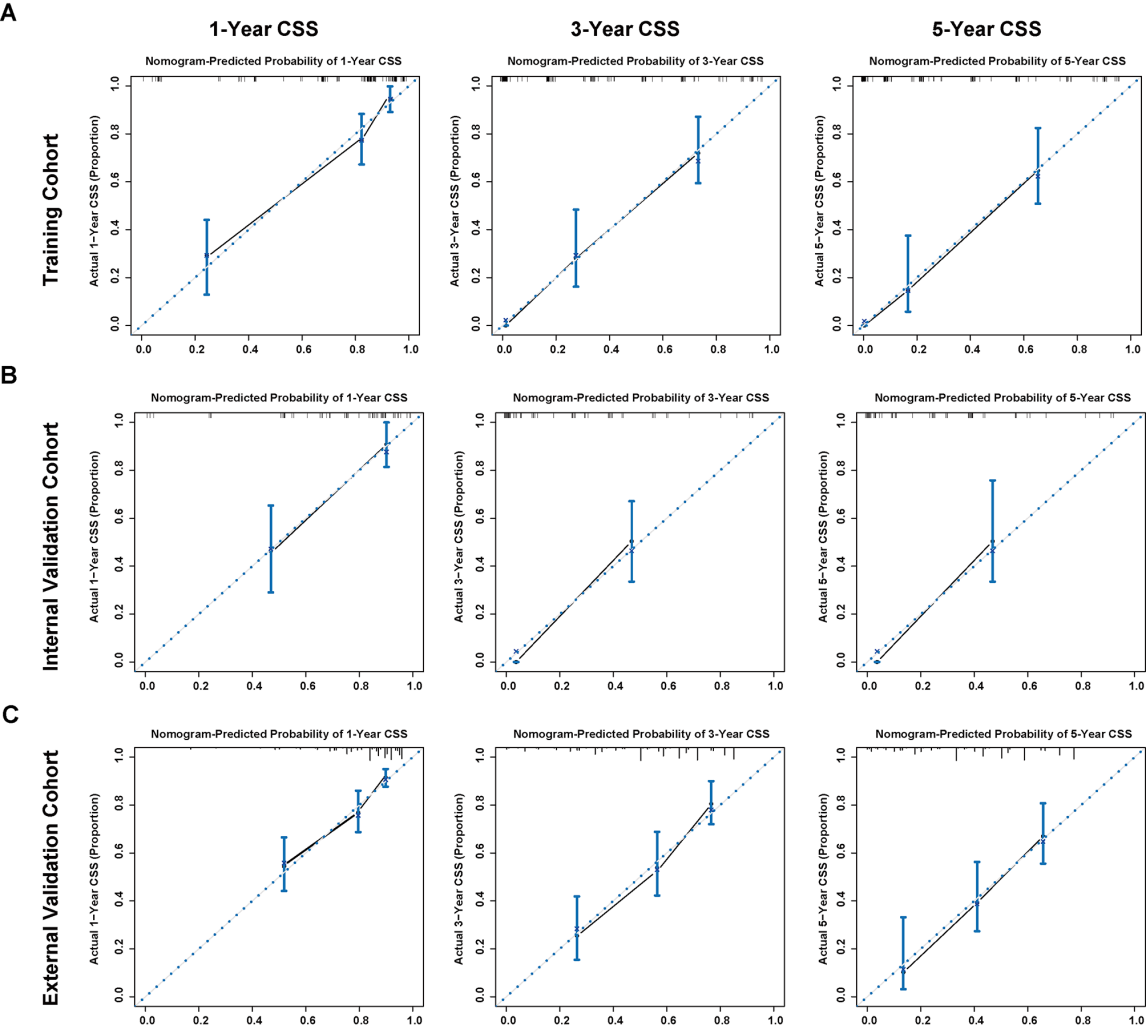
The nomogram’s calibration curves for the probability of 1-, 3-, and 5-year cancer-specific survival (**A**) The calibration curves for the probability of 1-, 3-, and 5-year cancer-specific survival in the training cohort; (**B**) The calibration curves for the probability of 1-, 3-, and 5-year cancer-specific survival in the internal validation cohort; (**C**) The calibration curves for the probability of 1-, 3-, and 5-year cancer-specific survival in the external validation cohort. Dashed lines represent the actual survival and solid lines represent the predicted survival

The C-index of our CCA nomogram for estimating CSS is 0.84 (95% CI, 0.80–0.88). Predicted and actual survival rates for cancer patients at 1, 3, and 5 years after radical resection were highly congruent, demonstrating a calibration curve (Fig. 3A). As showcased, there were shallow angles between the actual survival line and the predicted survival curve, which indicates there exists strong coherence between them. The estimated CSS over 1, 3, and 5 years may be quickly and readily determined by illustrating a straight line from the points where the scores for each variable sum up to the total scale. Based on this, it was glaringly obvious that the higher the total point of the nomogram showed, the poorer prognosis of CCA patients was.

### Comparison of the innovative nomogram and standard staging methods for predicting CCA cancer survival

**Fig. 4:**
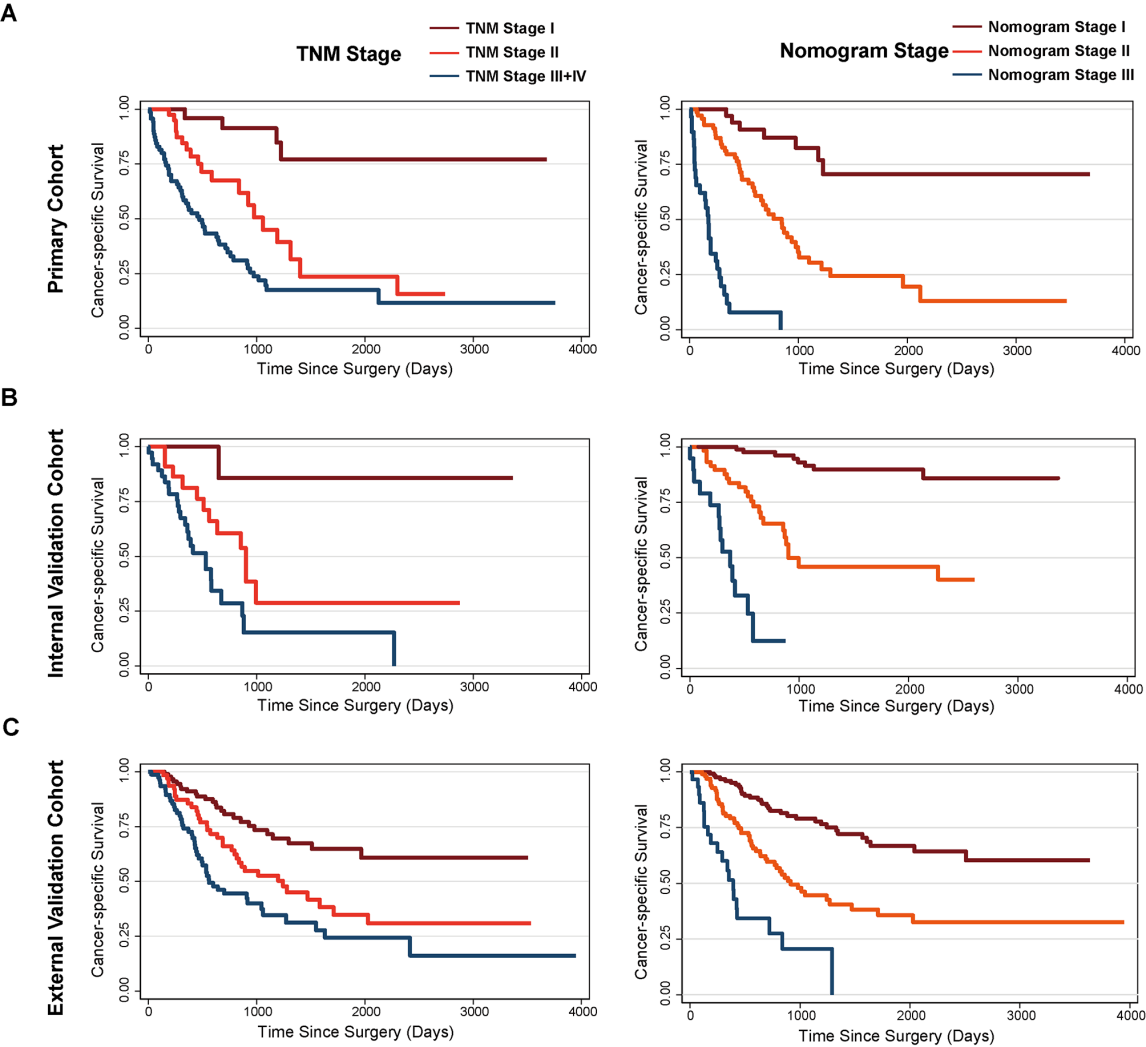
The prognostic significance of the TNM stage and our nomogram in the (**A**) training cohort, (**B**) internal, and (**C**) external validation cohort. The nomogram stage was defined by the total point value calculated by the nomogram: stage I (0-150), stage II (151–300), and stage III (>301)

**Fig. 5:**
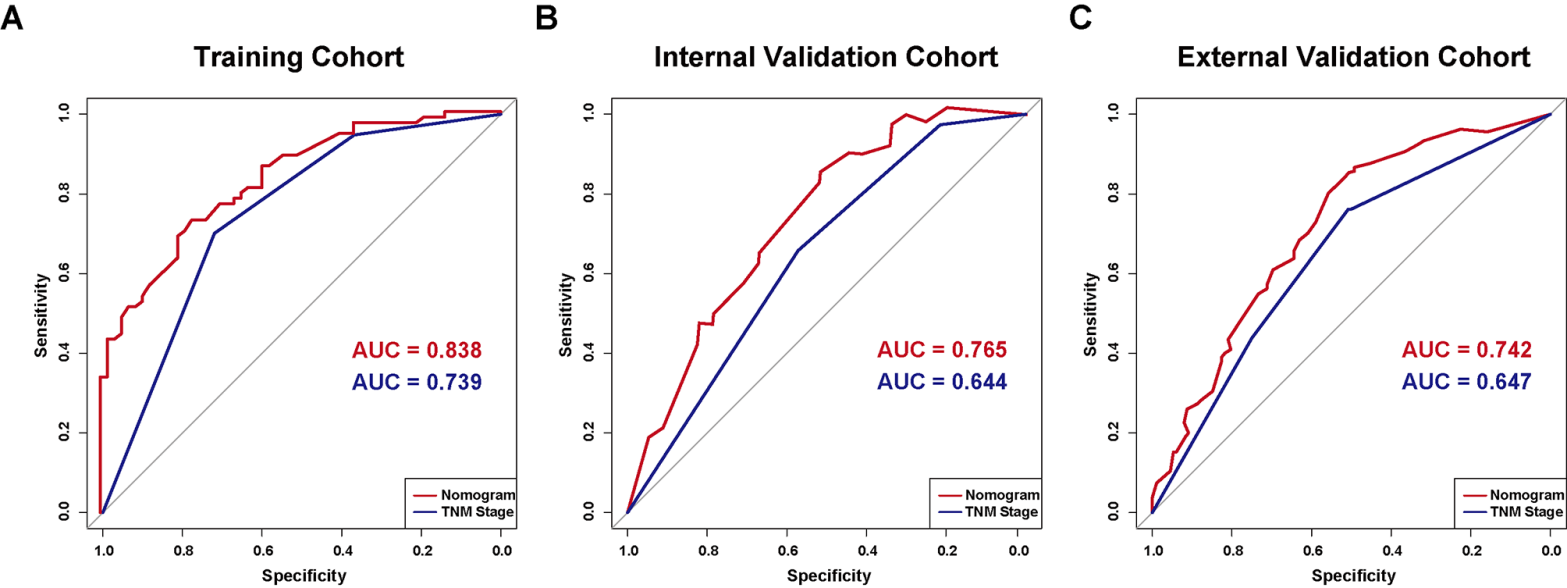
The nomogram and TNM stage time-dependent ROC curves for cancer-specific survival in CCA patients having radical surgery (**A**) The ROC curves for predicting CSS in the training cohort; (**B**) The ROC curves for predicting CSS in the internal validation cohort; (**C**) The ROC curves for predicting CSS in the external validation cohort. Area under the receiver operating characteristic curve, abbreviated as AUC

**Fig. 6:**
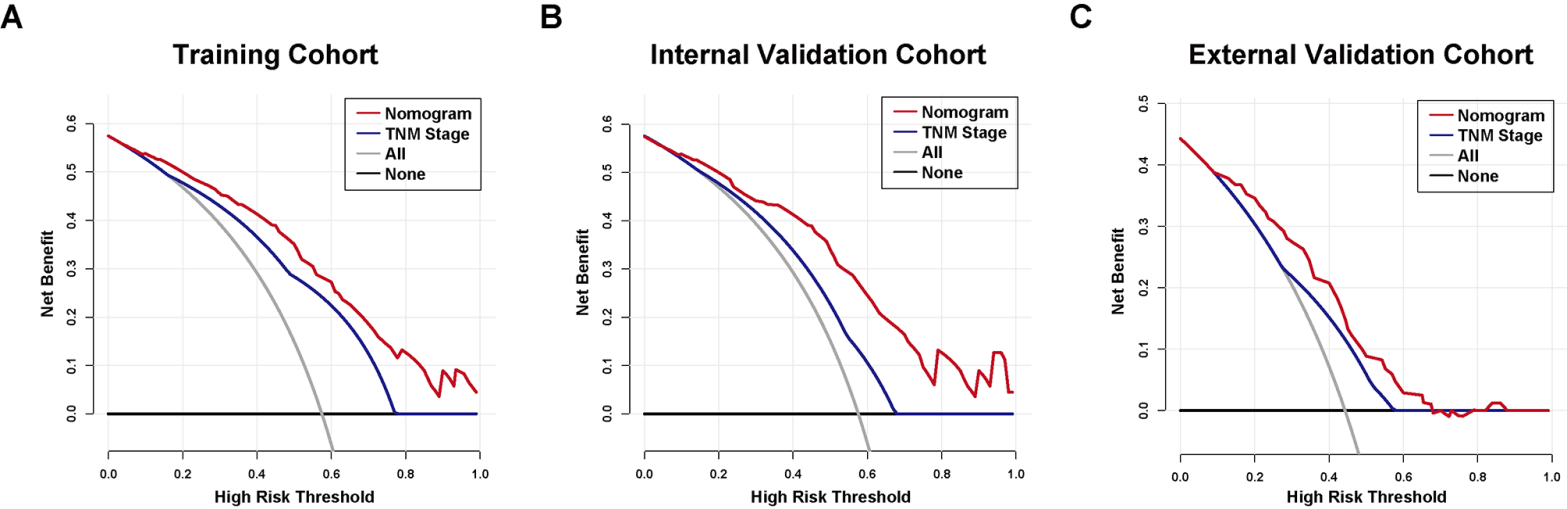
The decision curve analysis (DCA) of the nomogram and TNM stage for CCA patients receiving radical surgery for cancer-specific survival (**A**) DCA curve for predicting CSS in the training cohort; (**B**) DCA curve for predicting CSS in the internal validation cohort; (**C**) DCA curve for predicting CSS survival in the external validation cohort. Area under the receiver operating characteristic curve, abbreviated as AUC.

Using the C-index and time-dependent ROC curves, here, we compared the TNM staging system to the novel prognostic nomogram in terms of their discriminatory power. Both the short- and long-term survival predictions made by our technique and those made by the current staging method showed considerable improvements in the training and validation cohorts. The revised nomogram performed significantly better (P < 0.001) in the primary training cohort when the C-index of TNM stage was 0.74 (95%CI: 0.69–0.79). Our model revealed clearer prognostic strata than the conventional staging system, as indicated by Kaplan-Meier survival curves (Fig. 4A), especially for stage II and stage III. In addition, time-dependent ROC analysis was used to determine the prediction accuracy of the new nomogram (Fig. 5A) and decision curve analysis (Fig. 6A) to corroborate the aforementioned result. With respect to the standard staging approach for CCA patients’ chances of survival, the nomogram emerges as a more practical and accurate prognostic model.

### Internal validation of the CCA cancer-specific survival nomogram

From January 2009 to January 2019, 66 patients were enrolled in the same hospital as the training set to serve as an internal validation set. Table 1 displays the clinicopathologic features of patients. 45.9 months was the median follow-up period for the group used for internal validation (range, 9.8 to 130.0 months). CSS lasted a median of 23.3 months (range: 1.1-112.2 months), with the majority of cases lasting 1 year (63.6%), followed by 3 years (13.6%), and then 5 years (7.6%). In addition, our internal validation cohort demonstrated that our prognostic model was superior to the conventional TNM technique in predicting postoperative survival. The C-index of the nomogram was significantly higher (P < 0.001) than the AJCC-TNM 8th edition’s (0.65, 95%CI 0.58–0.73), and the calibration curves for the probability of 1-, 3-, and 5-year CSS showed reasonably great agreement between expected) values and the observed values (Fig. 3B). Figures 5B and 6B show ROC and DCA analyses for comparing the prediction performance of our nomogram to that of the aforementioned staging methods in the internal validation cohort. Furthermore, our model showed more distinct prognostic strata than did the conventional staging systems (Fig. 4B).

### External validation of the CCA cancer-specific survival nomogram

The external validation cohort included 235 CCA patients who had had a curative resection and were enrolled at the Sun Yat-sen University Cancer Center between January 2009 to January 2019. Table 1 displays the pertinent data for these patients. The median amount of time patients were followed up for in the external validation cohort was 40.8 months (range, 9.2 to 130.7 months). In addition, the CSS rates after 1 year were 77.1%, after 3 years were 31.5%, and after 5 years were 14.4%. The median time spent in CSS was 31.9 months (range, 1.1 to 131.6 months). When compared to the AJCC 8th editing staging system’s C-index of 0.65 (95% CI] 0.58–0.69), the nomogram’s C-index of 0.74 (95% CI] 0.70–0.79) was statistically significant (P < 0.001). One, three, and five-year survival probabilities predicted by the nomogram were shown to be in excellent agreement with actual observation along the calibration curve (Fig. 3C). To further confirm the more accurate prediction of nomogram than the usual TNM staging technique, Fig. 4 C, 5 C, and 6 C illustrate time-dependent ROC curves, Kaplan-Meier survival curves, and decision curve analysis (DCA) in the external validation cohort, respectively.

## Discussion

At present, surgical resection continues to be the cornerstone in the treatment of CCA patients [4, 25]. However, many CCA patients also experience poor prognoses even after undergoing tumor resection [26, 27]. In the literature, several staging algorithms have been used for the stratification of cancer and the choice of treatment options. Although several prognostic models are available for predicting the survival of CCA patients [6, 28, 29], there is no consensus on the most suitable options for postoperative prognosis. Therefore, it is essential to clarify the pathological mechanisms of tumor progression and to identify adverse prognostic factors, which are closely related to the selection of postoperative adjunctive treatment methods.

Past evidence suggested that inflammation is the key step in tumor progression, which depends on the reciprocal relationship between the systemic inflammatory response and tumor micro-environment [11, 30]. Cancer cells not only promote inflammatory cell infiltration and activation but also increase proinflammatory cytokine synthesis. And systemic inflammatory cells can activate the major inflammatory signaling pathways, influence the tumor microenvironment, and facilitate tumor growth, migration, and differentiation. Notably, the inflammatory index may be a choice of prognostic predictors for malignant tumors, including CCA, since systemic inflammation reflects the tumor burden [31]. It was reported that the pretreatment PLT level indicates the level of inflammation within the tumor [32]. As for the specific mechanism between platelets and tumor cells, increased circulating PLTs or functional activation may lead to tumor proliferation and metastasis through PLT–tumor interaction. On the one hand, cancerous cells can promote PLT aggregation and stimulate PLT activation by the secretion of tumor-associated proteins and cytokines such as thromboxane A2 and adenosine diphosphate. On the other hand, PLTs make a difference in communication with tumor cells and integration of the tumor microenvironment, which is involved in tumor development and chemotherapy resistance [33]. PLT–tumor interaction is considered a crucial step in the process of hematogenous metastasis. There was obvious evidence of a connection between the high level of PLTs and poor survival in several cancers [34–36]. Similarly, the high levels of PLTs also correlated with decreased CSS in CCA patients in our study.

Furthermore, it has been reported repeatedly that serum tumor biomarker level has a great impact on survival in cancer patients [37]. Even though the adverse prognostic implications of increased CEA and AFP levels in CCA are now known, their role in the stratification of the disease and guiding treatment are still unclear. Qiang et al. illustrated AFP and CEA levels were important prognostic indicators in CCA patients and can provide prognosis and survival assessment for CCA patients [38]. Moro et al. incorporated CA19-9 and CEA into the traditional staging system to improve survival prediction [39]. As a kind of simple, readily available, and noninvasive marker, serum tumor markers may serve as a preferred option for postoperative survival prediction. It may be beneficial in enhancing predictive performance and guiding treatment decision-making in postoperative CCA patients.

In our nomogram, we combined seven variables (PLT, CEA, AFP, tumor location, tumor differentiation, lymph node metastasis, and chemotherapy) recognized from multivariate analysis with the TNM stage to optimize clinical practice. First, compared to the 8th edition AJCC staging system, the sensitivity and specificity of the novel nomogram established based on the level of systemic inflammation state and tumor markers is much higher, allowing for accurate prediction of short- and long-term outcomes in postoperative CCA patients. Second, we calibrated and discriminated the nomogram across multiple centers to assess its efficacy. Not only the internal but also external validation cohorts were recruited to avoid selection bias and determine its general applicability. The integrated validations further prove the clinical usefulness of our nomogram in different populations. Thirdly, moreover, nomograms have been regarded as adjuncts in the prognostic determination of many kinds of cancers, which could create a specific score to provide a more powerful and accurate predicting outcome. The prominent advantage of the nomogram is the utility to help clinicians optimize reasonably targeted antitumor therapy after surgery. An online dynamic version of our nomogram was constructed by performing DynNom in R to support clinical and research decision (Fig. 2). By inputting personalized clinical data, the webserver based on our nomogram can calculate predicted survival probability and generate visualized figures and tables dynamically. Relying on this scoring system, clinical examination and tumor characteristics may be used to provide customized predictions of 1-, 3-, and 5-year survival in CCA patients. More application of the novel prognostic nomogram is supposed to practice.

The novel nomogram aforementioned in our study has significant prognostic potential. Many studies have been devoted to incorporating multiple markers to improve prognostic accuracy as a single factor is difficult to assess cancer prognosis perfectly [40]. Little is known about preoperative predictive markers to predict cancer-specific survival outcomes of CCA patients undergoing initial surgery. Studies to date pay more attention to predicting the overall outcomes of patients with CCA [41, 42]. Thus, we constructed a new nomogram by integrating inflammatory indicators, tumor markers, and other clinicopathological prognostic factors, which gave rise to the elevated c-index of 0.838 in comparison with 0.739 using the TNM stage alone to predict CSS. And this conclusion has also been verified in both internal and external validation cohorts. This novel dynamic model may assist in preoperative risk stratification, postoperative therapeutic intervention, and individualized surveillance strategies. Additionally, these promising findings provide a convenient approach to better identifying patients who may not take advantage of surgery. Patients with high biologic risk may need to choose or combine other kinds of therapy such as adjuvant or neoadjuvant therapy to improve prognosis and optimize economic benefit [43]. Despite the strengths of our current study, there are several limitations such as the retrospective study design. To achieve more reliable conclusions, more prospective research remains needed in the future.

## Conclusion

In conclusion, to predict the postoperative prognosis of CCA patients, the dynamic nomogram that includes serum markers and tumor signs is a viable and useful approach. Regarding the therapeutic schedule in postoperative decision-making, the nomogram as proposed in this study will be very useful for clinical decision-making and treatment optimization.

## Electronic supplementary material

Below is the link to the electronic supplementary material.

Supplementary Material 1

## Data Availability

The datasets generated during and/or analyzed during the current study are available from the corresponding author upon reasonable request.
