# Supplementary figures and images for: Cancer-specific survival in patients with cholangiocarcinoma after radical surgery: a Novel, dynamic nomogram based on clinicopathological features and serum markers
Source: BMC Cancer. 2023 Jun 12;23:533. doi: 10.1186/s12885-023-11040-9 (PMC10259060; doi:10.1186/s12885-023-11040-9)

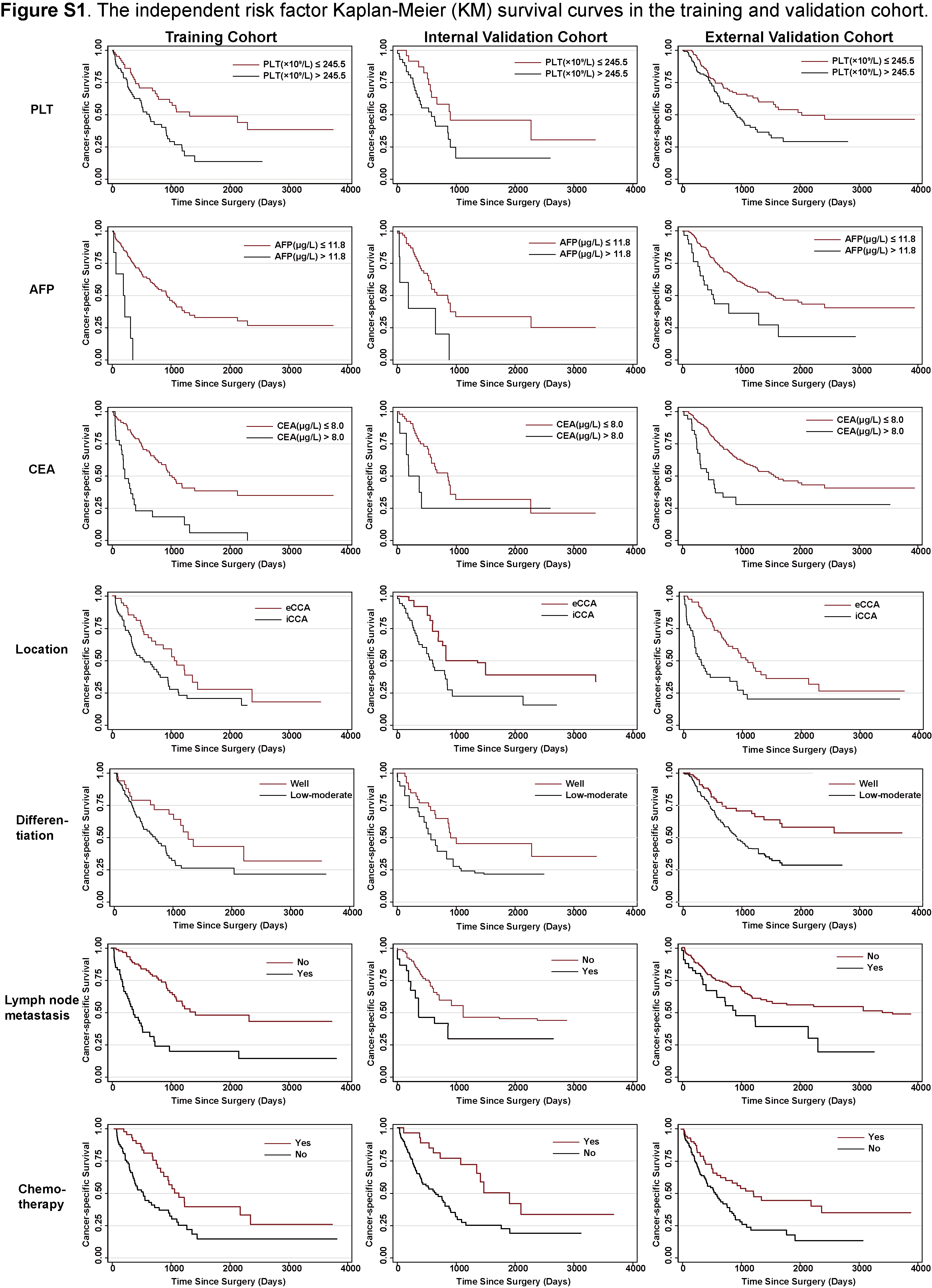

Supplement: Supplementary file 1 — Supplementary Material 1 [file 12885_2023_11040_MOESM1_ESM.png]
